# *α*-Glucosidase Inhibitory Constituents from *Acanthopanax senticosus* Harm Leaves

**DOI:** 10.3390/molecules17066269

**Published:** 2012-05-25

**Authors:** Zhi-Bin Wang, Hai Jiang, Yong-Gang Xia, Bing-You Yang, Hai-Xue Kuang

**Affiliations:** Key Laboratory of Chinese Materia Medica (Ministry of Education), Heilongjiang University of Chinese Medicine, Harbin 150040, China; Email: wzbmailbox@126.com (Z.-B.W.)

**Keywords:** *Acanthopanax senticosus* Harms, triterpene glycoside, alkaloid, *α*-glucosidase inhibition activity

## Abstract

A new triterpene glycoside, 3-*O*-[(*α*-L-rhamnopyranosyl)(1→2)]-[*β*-D-glucuronopyranosyl-6-*O*-methyl ester]-olean-12-ene-28-olic acid (**1**) and a new indole alkaloid, 5-methoxy-2-oxoindolin-3-acetic acid methyl ester (**5**) were isolated from the leaves of *Acanthopanax senticosus* Harms along with six known compounds. The structures of the new compounds were determined by means of 2D-NMR experiments and chemical methods. All the isolated compounds were evaluated for their glycosidase inhibition activities and compound **6** showed significant *α*-glucosidase inhibition activity.

## 1. Introduction

**Figure 1:**
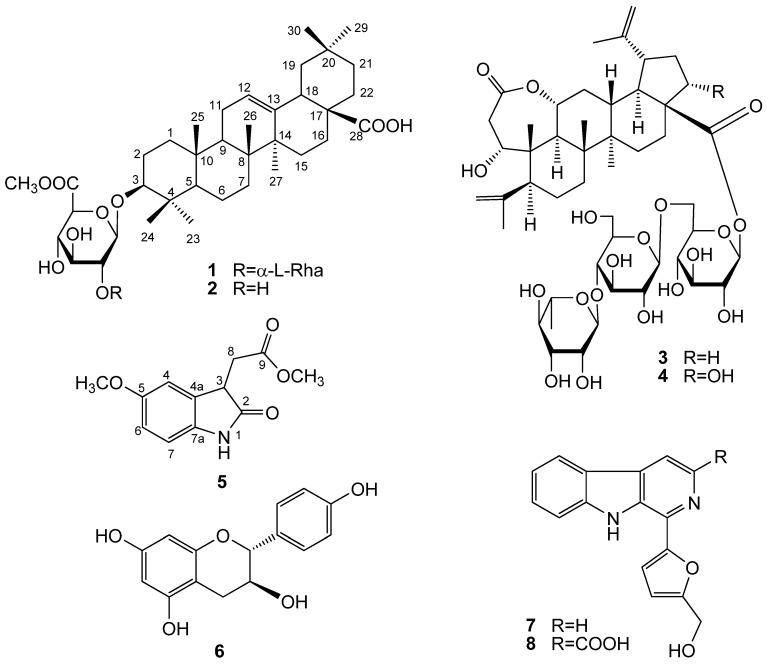
Structures of compounds **1**–**8**.

*Acanthopanax senticosus* (Rupr. Maxim) Harms, a member of the Araliaceae family, is a shrub found mainly in the northeast of China, Korea and Japan. It usually grows up to 2 m in height and generally has prickly stems bearing five leaflets (palmate) and umbel-shaped flowers. The young leaves of *A. senticosus* are cultivated as a vegetable crop in Northeast China. As regards biological active constituents of *A. senticosus*, many of its triterpenoid saponins [1,2,3,4] were reported to have anti-inflammatory [5], antibacterial [6] and platelet anti-aggregating activities [7]. Our studies indicated that the 30% EtOH fraction of *A. senticosus* leaves can inhibit plasma glucose levels in alloxan-induced diabetic rats and showed significant *α*-glucosidase inhibition activity. To further investigate the constituents and screen the bioactive compounds from its leaves, a phytochemical study was performed that resulted in the isolation of new compounds **1** and **5** along with six other previously known compounds. In this paper, we describe the structural elucidation of new compounds **1** and **5**, together with the *α*-glucosidase inhibitory activity of all these compounds **1**–**8** (Figure 1).

## 2. Results and Discussion

Compound **1** was obtained as a white amorphous powder (MeOH). The molecular formula was determined as C_43_H_68_O_12_ from the [M+Na]^+^ signal at *m/z* 799.4605 by HR-ESI-MS (calc. for C_43_H_68_O_12_Na 799.4608). A close comparison of NMR data (Table 1) of **1** with those of a known compound, oleanolic acid [3,8,9], indicated that the two compounds were very similar, except for two additional sugar units in **1**. By comparison of the ^13^C-NMR spectral data of **1** with those of oleanolic acid, the signal due to C-3 shifted to downfield by approximately 10 ppm, which indicated the sugar unit was linked to C-3 in **1**. On acid hydrolysis, **1** yielded L-rhamnose and D-glucuronic acid as a sugar component by GC analysis using a hydrogen flame detector after treatment with L-cysteine methyl ester hydrochloride in pyridine [9] and oleanolic acid was obtained and identified as the aglycone from NMR data by comparison with authentic samples. 

**Figure 2:**
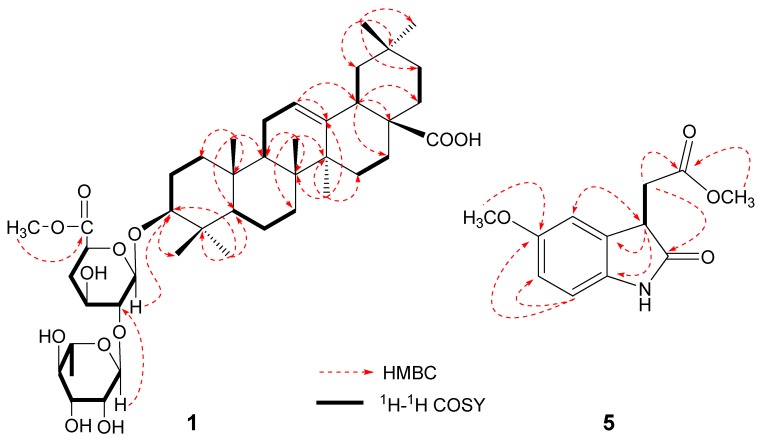
Key HMBC and ^1^H-^1^H COSY correlations of **1** and **5**.

The ^1^H-NMR spectrum of **1** (Table 1) showed seven methyl singlets *δ* 0.81 (3H), 0.94 (3H), 0.97 (3H), 1.00 (3H), 1.07 (3H) and 1.29 (6H), one olefinic H-atom (*br.s* at *δ* 5.45) and a methoxy group at *δ* 3.71 (3H). The^13^C-NMR spectrum of **1** showed signals of a pair of olefinic C-atoms at *δ* 122.5, 144.8, two anomeric C-atoms at *δ* 102.5, 105.4, and two C=O groups at *δ* 180.2 and 170.5. The ^1^H- and ^13^C-NMR signals of **1** were assigned by the use of HMQC and HSQC-TOCSY. The full connectivity of **1** was deduced from the HMBC correlations (Figure 2). In particular, the long-range correlations between the proton signals at *δ* 5.64 (H-1′′) and the carbon signals at *δ* 83.4 (C-2′) suggested that *α*-L-rhamnose unit was linked to C-2′ of *β*-D-glucuronic acid. The cross-peak between the proton signals at *δ* 3.71 (3H) and the carbon signals at *δ* 170.5 suggested that a methoxy group was linked to C=O of D-glucuronic acid. The anomeric configuration of D-glucuronic acid was determined to be *β*-form on the basis of the *J*_H-H_ value (7.6 Hz) in the ^1^H-NMR spectrum, and the configuration of L-rhamnose was determined to be *α*-form from examination of the chemical shifts (102.5, 72.4, 72.3, 73.7, 70.2, 18.2) in the ^13^C-NMR spectrum of **1**. Based on the above evidence, compound **1** was identified as 3-*O*-[(*α*-L-rhamnopyranosyl)(1→2)]-[*β*-D-glucuronopyranosyl-6-*O*-methyl ester]-olean-12-ene-28-olic acid.

**Table 1:** ^1^H and ^13^C-NMR data for compound **1** in pyridine-*d_5_*. (*δ* in ppm, *J* in Hz, recorded at 400 MHz and 100 MHz, respectively).

No.	*δ*_H_	*δ*_C_	No.	*δ*_H_	*δ*_C_
1	0.89 (1H, m); 1.50 (1H, m)	38.6	24	1.07 (3H, s)	16.4
2	1.84 (1H, m); 2.07 (1H, m)	26.6	25	0.81 (3H, s)	15.5
3	3.26 (1H, dd, 11.5, 4.0)	89.2	26	0.97 (3H, s)	17.4
4		39.5	27	1.29 (3H, s)	26.2
5	0.72 (1H, m)	55.7	28		180.2
6	1.29 (1H, m); 1.43 (1H, m)	18.4	29	0.94 (3H, s)	33.4
7	1.31 (1H, m); 1.45 (1H, m)	33.2	30	1.00 (3H, s)	23.8
8		39.7	GluA-Me		
9	1.60 (1H, m)	47.9	1′	4.99 (1H, d, 7.6)	105.4
10		36.9	2′	4.16 (1H, m)	83.4
11	1.87 (2H, m)	23.7	3′	4.31 ^a^	77.4
12	5.45 (1H, br s)	122.5	4′	4.62 ^a^	73.8
13		144.8	5′	4.50 ^a^	76.8
14		42.2	6′		170.5
15	1.15 (1H, m); 2.30 (1H, m)	28.2	OMe	3.71 (3H, s)	52.1
16	1.96 (1H, m); 2.09 (1H, m)	23.7	Rha		
17		46.6	1′′	5.64 (1H, br s)	102.5
18	3.36 (1H, dd, 4.5, 14.0)	41.9	2′′	4.62 ^a^	72.4
19	1.78 (1H, m); 1.19 ^a^	46.4	3′′	4.50 ^a^	72.3
20		30.9	4′′	4.31 ^a^	73.7
21	1.15 (1H, m); 1.36 (1H, m)	33.2	5′′	4.85 (1H, m)	70.2
22	1.80 (1H, m); 1.94 (1H, m)	33.1	6′′	1.71 (3H, d, 6.3)	18.2
23	1.29 (3H, s)	27.8			

^a^ Overlapped signals.

Compound **5** was obtained as a yellow oil. The molecular formula C_12_H_13_NO_4_ was defined by HR-ESI-MS (*m/z*: 258.0740 [M+Na]^+^, calc. for C_12_H_13_NO_4_Na 258.0742). The ^1^H-NMR (Table 2) spectra showed the presence of an aromatic ring with an ABX-coupling system at *δ* 6.64 (1H, dd, *J* = 8.5, 2.4 Hz), 6.71 (1H, d, *J* = 8.5 Hz) and 6.73 (1H, d, *J* = 2.4 Hz). The signals at *δ* 3.83 (3H, s) and 3.67 (3H, s) were assigned to proton signals of methoxy group. The ^13^C-NMR spectrum data (Table 2) showed 12 C-atom signals including an aromatic ring *δ* (C) 108.4, 110.0, 115.3, 131.6, 135.9 and 154.4 and two C=O groups at *δ* (C) 181.1 and 173.2. 

**Table 2:** ^1^H and ^13^C-NMR data for compound **5** in CD_3_OD. (*δ* in ppm, *J* in Hz, recorded at 400 MHz and 100 MHz, respectively).

No.	*δ*_H_	*δ*_C_	No.	*δ*_H_	*δ*_C_
2		181.1	8	3.05 (1H, dd, 17.2, 4.8)	35.3
3	3.70 (1H, dd, 7.6, 4.8)	44.2		2.78 (1H, dd, 17.2, 7.6)	
4	6.73 (1H, d, 2.4)	110.0	9		173.2
5		154.4	4a		131.6
6	6.64 (1H, dd, 8.5, 2.4)	115.3	7a		135.9
7	6.71 (1H, d, 8.5)	108.4	5-OMe	3.83 (3H, s)	55.8
			-COOMe	3.67 (3H, s)	52.4

The ^1^H-^1^H COSY spectrum showed correlations between *δ* 3.05 (1H, dd, *J* = 17.2, 4.8 Hz), 2.78 (1H, dd, *J* = 17.2, 7.6 Hz) and *δ* 0.70 (1H, dd, *J* = 7.6, 4.8 Hz), suggesting a –CHCH_2_– fragment. Moreover, in the HMBC spectrum, the long-range correlations between the proton signals at *δ* 3.70 (H-3) and the carbon signals at *δ* 110.0 (C-4), 131.6 (C-4a), 135.9 (C-7a), 181.1 (C-2) and 173.2 (C-9) could be identified. Correlations were observed between the proton signals at *δ* 3.05 (H-8) and the carbon signals at *δ* 131.6 (C-4a), 181.1 (C-2) and 173.2 (C-9) (Figure 2). Thus, the structure of compound **5** could be elucidated as 5-methoxy-2-oxoindolin-3-acetic acid methyl ester. The absolute configuration of the 3-position in compound **5** has not been established yet.

The other compounds were characterized as 3-*O*-(*β*-D-glucuronopyranosyl-6-*O*-methyl ester)-olean-12-ene-28-olic acid (**2**) [10], chiisanoside (**3**), 22*α*-hydroxychiisanoside (**4**) [11], (+)-afzelechin (**6**) [12], perlolyrine (**7**) [13], 3-carboxylperlolyrine (**8**) [14] by comparing their NMR spectroscopic data with literature values. All these known compounds are reported for the first time in *A. senticosus* leaves.

We evaluated the isolated compounds for their inhibitory activity against *α*-glucosidase since some compounds are known *α*-glucosidase inhibitors [15]. The results are shown in Table 3, with acarbose used as a positive control. Compounds **1**, **4** and **6** exhibited *α*-glucosidase inhibitory activities with IC_50_ values of 186.0 μM, 908.5 μM and 819.7 μM, respectively. Thus, this research suggested that the saponins and (+)-afzelechin from 30% EtOH fraction were primary *α*-glucosidase inhibitors.

**Table 3:** *In vitro α*-glucosidase inhibitory assay.

Compound	IC_50_ (μM ± SEM, μM)
**1**	908.5 ± 67.29
**2**	NI
**3**	NI
**4**	819.7 ± 91.61
**5**	NI
**6**	186.0 ± 12.01
**7**	NI
**8**	NI
Acarbose	788.6 ± 53.66

NI no inhibition at 1,000 µM concentration.

## 3. Experimental

### 3.1. General

Open column chromatography was carried out using silica gel (200–300 mesh, Qingdao Marine Chemical Co., Qingdao, China) or octadecyl silica gel (ODS, 25–40 μm, Fuji, Tokyo, Japan) as stationary phases. TLC employed precoated silica gel plates (5–7 μm, Qingdao Marine Chemical Co., Qingdao, China). Preparative HPLC was carried out on a Waters 600 instument equipped with a Waters RID-2414 detector. A Waters Sunfire prep C_18_ OBD (19 × 250 mm i.d.; Milford, PA, USA) column was used for preparative purposes. The IR spectra were recorded as KBr pellets on a Jasco 302-A spectrometer. Optical rotation was recorded on a Jasco P-2000 polarimeter. HRESIMS were measured on a FTMS-7 instrument (Bruker Daltonics, Karlsruhe, Germany). Melting points were determined on a Gallenkemp apparatus and are uncorrected. The ^1^H-, ^13^C- and 2D (^1^H-^1^H COSY, HSQC, HMBC, NOESY) NMR spectra were recorded on a Bruker AMX-400 spectrometer using standard pulse sequences. Chemical shifts are reported in ppm (*δ*), and scalar coupling are reported in Hz. GC analyses were carried out using a Fuli 9790 instrument, DM-5 column (0.25 μm, 30 m × 0.25 mm, Dikma, Beijing, China). *α*-Glucosidase (EC.3.2.1.20) from *Saccharomyces* sp. was purchased from Wako Pure Chemical Indutries Ltd. (Wako 076-02841; Osaka, Japan). 

### 3.2. Plant

The leaves of *A. senticosus* Harms were collected in October 2009 from Fangzheng of Heilongjiang Province, China and identified by Prof. Zhenyue Wang, of Heilongjiang University of Chinese Medicine. A voucher specimen (20090929) was deposited at the herbarium of Heilongjiang University of Chinese Medicine, Harbin, China.

### 3.3. Extraction and Isolation

Young leaves (6 kg) of *A*. *senticosus* Harms was extracted with 70% EtOH (3 × 48 L) under reflux. The combined extract was concentrated under vacuum yielding a residue (580 g) which was dissolved in water, loaded on a D101 macroporous adsorption resin column and eluted successively with H_2_O, 30% EtOH, 60% EtOH and 95% EtOH. The 30% EtOH fraction (80.0 g) was subjected to silica gel column chromatography with a stepwise CHCl_3_-MeOH gradient (10:1, 5:1, 3:1, 2:1 v/v), and finally with MeOH alone, to give five fractions (I–V). Fraction II (5.25 g) was rechromatographed over SiO_2_ with CHCl_3_/MeOH/H_2_O (5:1:0.1) and separated further by HPLC using a C_18_ OBD column (10 μm, 19 × 250 mm, flow rate 10 mL/min) with MeOH/H_2_O 15:85 to give **5** (30.5 mg; *t*_R_ 7.0 min), **6** (22.1 mg; *t*_R_ 12.5 min) and **7** (11.8 mg; *t*_R_ 22.1 min). Fraction III was subjected to reversed-phase silica gel column chromatography [10 g, MeOH/H_2_O (20:80→30:70→40:60→50:50→60:40→70:30, v/v)→MeOH] to afford six fractions [Fr. III_1_, Fr. III_2_, Fr. III_3_, Fr. III_4_, Fr. III_5_, Fr. III_6_). Fr. III_3_ was separated by HPLC [MeOH/H_2_O (50:50, v/v)] to give **2** (23.4 mg *t*_R_ = 43.2 min). Fr. III_4_ was separated by HPLC [MeOH/H_2_O (55:45, v/v)] to give **8** (35.3 mg, *t*_R_ =12.7 min) and **1** (10.1 mg, *t*_R_ =17.3 min). Fr. III_5_ was separated by HPLC [MeOH/H_2_O (53:47, v/v)] to give **3** (12.7 mg, *t*_R_ =22.1 min). Fraction IV was subjected to reversed-phase silica gel column chromatography [10 g, MeOH/H_2_O (30:70→40:60→ 50:50→60:40→70:30, v/v)→MeOH] to afford three fractions [Fr. IV_1_, Fr. IV_2_, Fr. IV_3_). Fr. IV_2_ was separated by HPLC [MeOH/H_2_O (48:52, v/v)] to give **4** (14.5 mg, *t*_R_ =28.1 min). 

### 3.4. Characterization Data

*3-O-[(α-L-Rhamnopyranosyl)(1→2)]-[β-D-glucuronopyranosyl-6-O-methyl ester]-olean-12-ene-28-olic acid* (**1**). White amorphous powder (MeOH). [*α*]^20^_D_ +10.5° (*c* 0.15, MeOH). IR (KBr): 3408, 2946, 1695, 1387, 1159, 1078, 637 cm^−1^. HR-ESI-MS *m/z* 799.4605 [M+Na]^+^ (calc. C_43_H_68_O_1__2_Na, 799.4608); ^1^H and ^13^C-NMR (pyridine-*d_5_*) data, see Table 1.

*5-Methoxy-2-oxoindolin-3-acetic acid methyl ester* (**5**). Yellow oil (MeOH). [*α*]^20^_D_ +4.5° (*c* 0.25, MeOH). UV *λ* MeOH max nm (log *ε*): 304 (3.11), 257 (3.76), 208 (4.05); IR (KBr): 3376, 2951, 1700, 1615, 1293, 1255, 1091 cm^−1^. HR-ESI-MS *m/z* 258.0740 [M+Na]^+^ (calc. C_12_H_13_NO_4_Na, 258.0742); ^1^H and ^13^C-NMR (CD_3_OD) data, see Table 2.

### 3.5. Acid Hydrolysis of ***1***

Compound **1** (3 mg) was hydrolyzed in 1 M HCl (1.0 mL) for 2 h at 85°. The reaction mixture was cooled and partitioned between CHCl_3_ (2.0 mL) and H_2_O (2.0 mL). The aqueous layer was washed with CHCl_3_ (3 × 3.0 mL), neutralized with Ba(OH)_2_, filtered, and evaporated under reduced pressure. The residue was dissolved in pyridine (1.0 mL) and L-cysteine methyl ester hydrochloride in pyridine (0.1 M, 2.0 mL) was added. The mixture was heated at 60° for 1 h. An equal volume of Ac_2_O was added with heating and incubation was continued for 1 h. The acetylated thiazolidine derivatives were analyzed by GC with a DM-5 column (30 m × 0.25 mm, 0.25 μm) using authentic samples as standards. Temperatures of both injector and detector were 280°. A temperature gradient system was used for the oven, starting at 160° and increasing up to 195° at a rate of 5°/min. The determination of the sugar units in compound **1** was achieved by comparison of retention times of peaks between the hydrolysate and authentic samples of D-glucuronic acid (10.08 min) and L-rhamnose (7.05 min). 

### 3.6. α-Glucosidase Inhibition Assay

The *α*-glucosidase (EC.3.2.1.20) enzyme inhibition assay has been performed according to the literature [16]. *α*-Glucosidase (25 μL, 0.2 U/mL), various concentrations of samples (25 μL), and 67 mM phosphate buffer (pH 6.8, 175 μL) were mixed at room temperature for 10 min. Reactions were initiated by the addition of 23.2 mM *p*-nitrophenyl-*α*-D-glucopyranoside (25 μL). The reaction mixtures were incubated at 37 °C for 15 min in a final volume of 250 μL, and then 1 M Na_2_CO_3_ (50 μL) was added to the incubation solution to stop the reaction. The activities of glucosidase were detected in a 96-well plate, and the absorbance was read at 405 nm by a microplate spectrophotometer (Spectra Max, Molecular Devices, Sunnyvale, CA, USA). The negative control was prepared by adding phosphate buffer instead of the sample in the same way as the test. Acarbose was utilized as the positive control. The blank was prepared by adding phosphate buffer instead of *α*-glucosidase using the same method. The inhibition rates (%) were calculated from the formula:



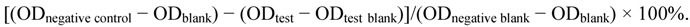



## 4. Conclusions

Two new compounds, 3-*O*-[(*α*-L-rhamnopyranosyl)(1→2)]-[*β*-D-glucuronopyranosyl-6-*O*-methyl ester]-olean-12-ene-28-olic acid (**1**) and 5-methoxy-2-oxoindolin-3-acetic acid methyl ester (**5**) were isolated from the 30% EtOH fraction of *A. senticosus* leaves, along with six known compounds. Compounds **1**, 22*α*-hydroxychiisanoside (**4**) and (+)-afzelechin (**6**) showed inhibitory activities against *α*-glucosidase, with IC_50_ values of 186.0 µM, 908.5 µM and 819.7 µM, respectively.

## References

[1] Jiang W., Li W., Han L., Liu L., Zhang Q., Zhang S., Nikaido T., Koike K. (2006). Biologically active triterpenoid saponins from *Acanthopanax senticosus*. J.Nat.Prod..

[2] Park S.Y., Chang S.Y., Yook C., Nohara T. (2000). New 3, 4-seco-lupane-type triterpene glycosides from *Acanthopanax senticosus* forma *inermis*. J. Nat.Prod..

[3] Shao C.J., Kasai R., Xu J.D., Tanka O. (1989). Saponins from leaves of *Acanthopanax senticosus* HARMS., Ciwujia.II. structures of Ciwujianosides A_1_, A_2_, A_3_, A_4_ and D_3_. Chem. Pharm. Bull..

[4] Shao C.J., Kasai R., Xu J.D., Tanka O. (1988). Saponins from leaves of *Acanthopanax senticosus* HARMS., Ciwujia: Structures of Ciwujianosides B, C_1_, C_2_, C_3_, C_4_, D_1_, D_2_ and E. Chem. Pharm. Bull..

[5] Jung H.J., Nam J.H., Choi J., Lee K.T., Park H.J. (2005). Antiinflammatory effects of chiisanoside and chiisanogenin obtained from the leaves of *Acanthopanax senticosus* in the carrageenan and Freund’s complete adjuvant-induced rats. J. Ethnopharmacol..

[6] Lee S., Shen D.S., Oh K.B., Shin K.H. (2006). Antibacterial compounds from the leaves of *Acanthopanax senticosus*. Arch. Pharm. Res..

[7] Jin J.L., Lee S., Lee Y.Y., Kim J.M., Heo J.E., Yun-Choi H.S. (2004). Platelet anti-aggregating triterpenoids from the leaves of *Acanthopanax senticosus* and the fruits of *A. sessiliflorus*. Planta Med..

[8] Song S.J., Nakamura N., Ma C.M., Hattori M., Xu S.X. (2001). Five saponins from the root bark of *Aralia elata*. Phytochemistry.

[9] Yang C.J., An Q., Xiong Z.L., Song Y., Yu K., Li F.M. (2009). Triterpenes from *Acanthopanax sessiliflorus* fruits and their antiplatelet aggregation activities. Planta Med..

[10] Melek F.R., El-gindi O.D., Abdel-Khalik S.M., Miyase T., Haggag M.Y. (1996). Saponins from *Fagonia mollis*. Phytochemistry.

[11] Shirasuna K., Miyakoshi M., Mimoto S., Isoda S., Satoh Y., Hirai Y., Ida Y., Shoji J. (1997). Lupane triterpenoid glycosyl esters from leaves of *Acanthopanax divaricatus*. Phytochemistry.

[12] Saijyo J., Suzuki Y., Okuno Y., Yamaki H., Suzuki T., Miyazawa M. (2008). *α*-Glucosidase inhibitor from *Bergenia ligulata*. J.Oleo Sci..

[13] Nakatsuka S.I., Feng B.N., Goto T., Kihara K. (1986). Structures of flazin and YS, highly fluorescent compounds isolated from *Japanese soy sauce*. Tetrahedron Lett..

[14] Su B.N., Chang L.C., Park E.J., Cuendet M., Santarsiero B.D., Mesecar A.D., Mehta R.G., Fong H.H.S., Pezzuto J.M., Kinghorn A.D. (2002). Bioactive constituents of the seeds of *Brucea javanica*. PlantaMed..

[15] Li W., Fu H., Bai H., Sasaki T., Kato H., Koike K. (2009). Triterpenoid saponins from *Rubus ellipticus* var. *obcordatus*. J. Nat. Prod..

[16] Kuang H.X., Li H.W., Wang Q.H., Yang B.Y., Wang Z.B., Xia Y.G. (2011). Triterpenoids from the roots of *Sanguisorba tenuifolia* var. Alba. Molecules.

